# ‘The problem is small enough, the problem is big enough’: a qualitative study of health technology assessment and public policy on drug funding decisions for children

**DOI:** 10.1186/s12939-020-01164-w

**Published:** 2020-03-30

**Authors:** Avram E. Denburg, Mita Giacomini, Wendy J. Ungar, Julia Abelson

**Affiliations:** 1 Division of Haematology/Oncology, Department of Paediatrics, The Hospital for Sick Children, 555 University Ave, Toronto, Ontario M5G 1X8 Canada; 2 Child Health Evaluative Sciences, Peter Gilgan Centre for Research and Learning, The Hospital for Sick Children, Toronto, Canada; 3 Institute of Health Policy, Management and Evaluation, University of Toronto, Toronto, Canada; 4 Centre for Health Economics and Policy Analysis, Department of Health Research Methods Evidence and Impact, McMaster University, Hamilton, Canada

**Keywords:** Canada, Health technology assessment, Drug policy, Children, Social values, Health system, Resource allocation, Priority setting

## Abstract

**Background:**

Public policy approaches to funding paediatric medicines in developed public health systems remain understudied. Current approaches to HTA present a variety of conceptual, methodological and practical problems in the context of child health. This study explores the technical and sociopolitical determinants of public funding decisions on paediatric drugs, through the analysis of interviews with stakeholders involved in or impacted by HTA for child health technologies at the provincial and national levels in Canada.

**Methods:**

We undertook in-depth interviews with a purposive sample (*n* = 22) of stakeholders involved with or affected by drug funding decisions for children at the provincial (Ontario) and national levels in Canada. Grounded theory methods were employed to guide data collection and analysis. Theory on ‘technology-as-policy’ and the sociopolitics of health technologies served as sensitizing concepts for inductive data coding and analysis. Emergent themes informed the development of conceptual and practical insights on social values and system dynamics related to child HTA, of relevance to public policymaking on the coverage of health technologies for children in Canada.

**Results:**

Participant reflection on the normative and systems dimensions of drug funding for children formed two broad categories: HTA paradigms and sociopolitical context. Our analysis revealed notable differences of context and substance related to child health technology production, evaluation and use. These differences spanned the major phases of HTA (from assembly to assessment to integration) and the surrounding sociopolitical milieu (from markets to governance to politics). Careful analysis of these differences sets in relief a number of substantive and procedural shortcomings of current HTA paradigms in respect of child health. Our findings suggest a need to rethink how HTA is structured and operationalized for child health technologies.

**Conclusions:**

Current approaches to health technology assessment are not well calibrated to the realities of child health and illness. Our study presents a nuanced and contextually grounded analysis of concepts instrumental to drug funding decisions for children. The insights generated are directly applicable to the Canadian and Ontario contexts, but also yield fundamental knowledge about HTA for children that are germane to drug policy in other health systems.

## Background

Despite advances over recent decades in child-specific drug regulatory provisions for drug approval in the United States (US) and European Union (EU), public policies on the *funding* of paediatric medicines in developed health systems have garnered little attention [1–3]. Health technology assessment (HTA) frameworks appraise the value of ‘technologies’ – drugs, devices, procedures or services – to inform policy decision-making and resource allocation within publicly funded health systems [4]. Decisions about whether or not to allocate public funds to cover the costs of novel drugs or diagnostics is increasingly the purview of regional or national HTA institutions in most developed countries. There is growing awareness that current approaches to HTA present a variety of problems in the context of child health. These range from methodological issues, including standard criteria for evidence appraisal and health economic evaluation, to system ones, including how technologies are prioritized for review and adjudicated for public funding [5, 6].

**Fig. 1:**
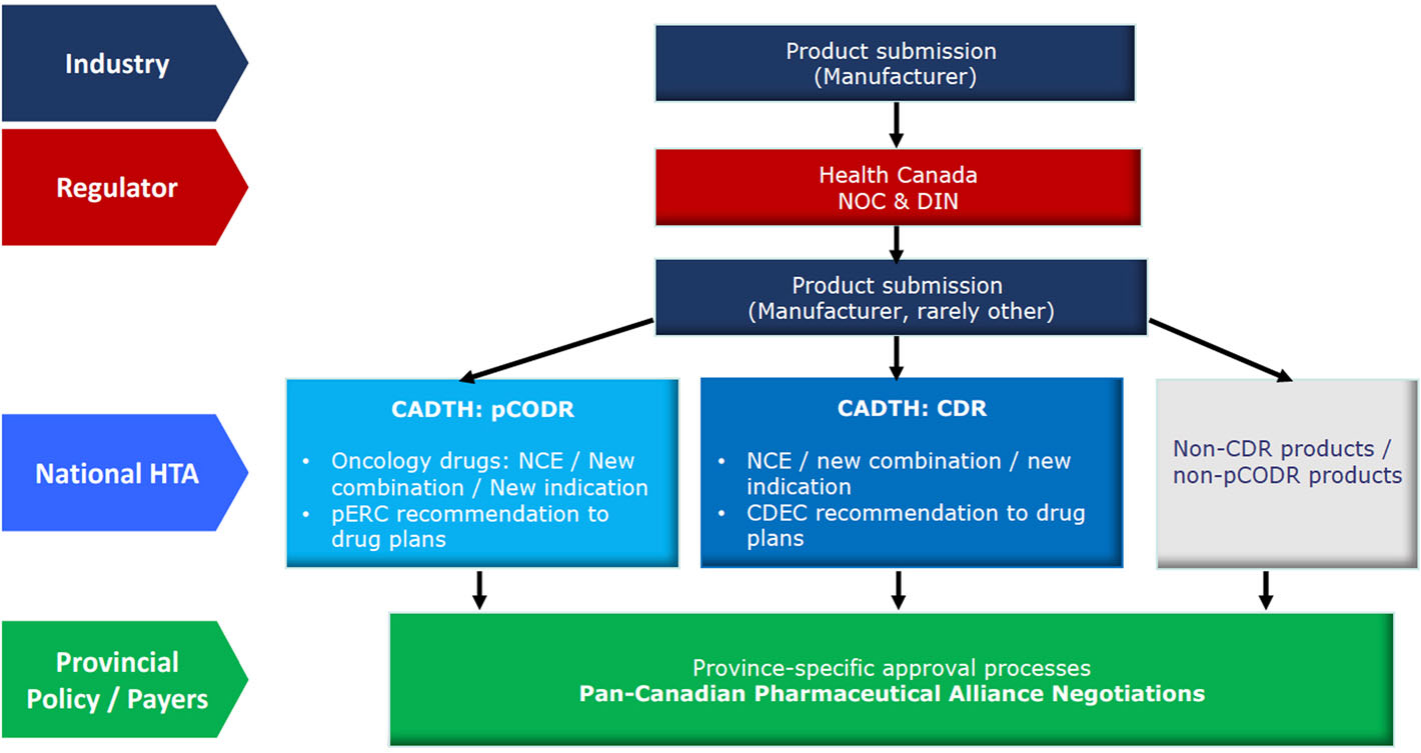
Drug approval and funding process in Canada (except Quebec)

HTA plays a critical role in drug policy in an increasing number of developed health systems. Formal technology assessment is now a standard component of public drug coverage decisions in Canada. The Canadian Agency for Drugs and Technologies in Health (CADTH) conducts national-level HTA reviews for novel drugs and therapeutics licensed for sale in Canada by the federal drug regulator, Health Canada [7]. Its recommendations are passed on to the provinces (with the exception of Quebec), where ultimate authority for public sector drug policy resides (Fig. 1). While some provincial authorities recapitulate CADTH’s reviews, most now rely on and use national technology assessments to inform their drug coverage decisions. Through the Pan-Canadian Pharmaceutical Alliance (PCPA), provinces are engaging in collective drug price negotiations with industry [8]. In the context of mounting cross-provincial engagement on drug policy, CADTH’s HTA recommendations play an increasingly important role in guiding cross-provincial policy harmonization.

The mounting importance of HTA in public drug funding decisions in Canada and comparable countries has exposed its awkward fit for the evaluation of paediatric health technologies. Child-focused health technologies are rarely prioritized for review; when they are, standard approaches to evaluating clinical evidence, cost effectiveness, and societal value are poorly adapted to the often-unique realities of child health and illness [6]. Almost no inquiry exists into the nature and extent of these shortcomings, the potential means of resolving them, and the resultant implications for policies on public drug coverage, in Canada or elsewhere. This article explores the sociopolitical and health system dynamics that influence decision-making for public funding of paediatric drugs, through analysis of interviews with stakeholders involved in or impacted by HTA for child health technologies at the provincial (Ontario) and national levels in Canada. It contributes novel data to inform the assessment and prioritization of funding for paediatric drugs and health technologies, with direct policy relevance to health care priority-setting bodies and government funders in Canada and internationally.

## Methods

### Data collection

Between December 2016 and April 2017, we undertook a series of in-depth, semi-structured interviews with a purposive sample (*n* = 22) of stakeholders involved with or affected by drug funding decisions for children. The sample included: parents of children with cancer and other chronic diseases (PAR; *n* = 4); health professionals (physicians, allied health, pharmacists, clinical bioethicists) involved in the care of such patients (HEA; *n* = 7); HTA professionals at the national level in Canada (PRO; *n* = 4); and provincial policymakers involved with drug coverage decisions in Ontario (POL; *n* = 7). We identified potential participants through grey literature review, institutional scans of relevant hospitals, HTA organizations and government websites, and snowball sampling. Study participation was voluntary, and all participants provided written informed consent prior to participating in interviews. Interviews were audiotaped, transcribed verbatim and inductively coded using NVivo 11 software (QSR International, Ltd.). Data were anonymized and de-identified to protect participant confidentiality. Ethics approval for this study was granted by the Hamilton Integrated Research Ethics Board affiliated with McMaster University.

### Data analysis

Using constructivist grounded theory methodology, we undertook iterative sequential phases of data coding, moving from open through theoretical codes, with constant comparative methods employed to refine codes, establish analytic distinctions, and capture emergent themes [9]. We conducted additional interviews to generate and refine salient themes, until theoretical saturation was reached. These themes informed the development of conceptual and practical insights on system dynamics related to child HTA, to inform public policymaking on the coverage of health technologies for children in Canada. We drew on theoretical conceptions of ‘technology-as-policy’ and the sociopolitics of health technologies as sensitizing concepts for our inductive coding and analysis of the data [10–14]. The notion of technology-as-policy invokes the inherently political nature of all technologies [14]. It underscores the power dynamics created and mediated by discrete technologies, and the moral implications of their development, use and disuse. Lehoux and Blume provide a normative framework to interrogate the sociopolitics of health technologies, which emphasizes four domains of assessment: actors, resources, knowledge, and power [13]. We employed this framework as an heuristic to critically analyze the concepts and themes generated from our data.

## Results

Participant reflection on the normative and systems dimensions of drug funding for children coalesced into two broad categories: HTA paradigms and sociopolitical context. Each of these categories subsumes multiple themes.

### HTA paradigms

Foundational ideas about the nature and role of technology in shaping health outcomes across the life-course emerged from participants’ reflection on, and challenges to, current approaches to the assessment of health technologies for children. These challenges spanned the productive phases of HTA: from the *assembly* and prioritization of technologies, through the component parts of formal *assessment*, to health system *integration*. In each of these phases, participants located points of poor fit between HTA process or structure and the realities of child health and illness; they also identified opportunities to better align HTA to these realities.

#### Assembly

The manner in which technologies are selected, packaged and presented for formal assessment by HTA institutions is an oft-overlooked but critical part of the process that determines public funding decisions for drugs and other health technologies [15, 16]. This *assembly* phase is generative: it frames the perceived place, use and relevance of a given technology in relation to others; in so doing, it conditions both the priority assigned a technology and the parameters for conducting its review [17]. A number of the HTA and health care professionals interviewed spoke to the importance of a birds’-eye view of the health system in setting up the trade-offs inherent in HTA. Emphasizing the opportunity costs of selecting technologies for assessment, these participants identified myopia in current HTA paradigms, in which decision-making is often abstracted from the wider health system context:

“*If we want to design and execute a health care system that is evidence-informed and reflects our values, then actually we also have to tackle those higher-level resource allocation decisions.*” (PRO3)

They emphasized the added relevance of system-level perspective in the prioritization of child health technologies, arguing that standard metrics of priority-setting in HTA may require rethinking to balance conventional concerns with those specific to the epidemiology and management of childhood disease. Disease burden as a principal determinant of HTA priority-setting was a notable example. One participant referenced the need for more nuanced priority-setting based on disease prevalence, noting:

“*If your view is you have to look first at the most common cancers, one hundred percent of the time children are going to be back of the queue.*” (POL2)

In aggregate, a clear concern emerged that the values incorporated into HTA priority-setting, and the assembly of health technologies more broadly, is insufficient. The corollary of this in the paediatric space was a recognition that better incorporation of relevant values could help rebalance priority-setting exercises to correct for an intrinsic bias against child health technologies, which we discuss in greater detail below.

In addition to a critique of the values that structure HTA assembly, participants also cited problems with the processes that condition it. Clinicians, HTA professionals and parents alike viewed the submission process for HTA review as routinely at odds with the nature of paediatric drug development and use. The reliance on industry to put forward drugs for review by national HTA bodies – and, notably, to generate and package the clinical and economic evidence that enables it – was seen to limit the scope of paediatric drugs evaluated for public funding. Participants located the root of this problem in the disjunct between industry profit incentives and the costs of doing business in paediatric drug development. Both the small size of childhood drug markets and the added complexity of clinical trials involving children conspire to deter industry from the work involved in submission for HTA review.

In the absence of legislative provisions in Canada to incentivize or compel industry to generate drug safety and efficacy data in children, there is little financial reason for companies to seek either market approval or public coverage for most paediatric medicines. This lack of motivation has outsized impact in an HTA system heavily reliant on manufacturer-initiated submissions. As a number of participants noted, it shifts the responsibility for submission to other health system stakeholders, such as clinicians and patient advocacy groups. These parties are often poorly resourced to undertake the complicated work of HTA submission, which involves extensive evidence review and pharmacoeconomic modelling. More fundamentally, weak industry interest in childhood drug research limits the very evidence upon which funding recommendations are made: pharmaceutical companies are less likely to invest in establishing firm paediatric indications for their products in the absence of compelling markets for their sale. The HTA professionals interviewed identified the established indications for drug use as a critical component of HTA assembly:

*“We typically only look at the Health Canada approved indications, so, therefore, if it has a restriction of 18 years of age or older…it's our understanding that when we put these recommendations forward, the payers are following the indication for use.”* (PRO4)

The relationship between market dynamics and evidence generation was a major theme voiced by participants, one with broader implications than on HTA assembly alone. We deal with this theme in greater detail below.

To better align HTA submissions with patient and health system needs, participants called for HTA institutions to do “more work at eliciting provider submissions, as opposed to everything coming from pharma” (Health professional), recognizing that this would necessitate dedicated resources. One argued for built-in requirements to incorporate paediatric-specific indications for agents submitted to national HTA bodies. Others identified the need for a ‘paediatric HTA watchdog organization’, tasked with regular review of the landscape of HTA submissions in Canada and support of decision-makers in paediatric-specific evidence collation and interpretation. Some identified the potential for reciprocal benefits in such a relationship, alluding to a role for the HTA system itself to help define paediatric drug research priorities.

Taken together, these comments reveal both a nuanced understanding of the upstream factors conditioning funding decisions on child health technologies, and an appreciation that their redress will demand system reform on distinct fronts. The attention paid by participants to the under-explored phase of HTA assembly suggests its relative importance in the context of childhood drug access, and the need to attend to it in attempts to optimize HTA systems for children.

#### Assessment

The phase of technology *assessment* received the most detailed reflection and scrutiny by study participants. Its component parts – evidence appraisal, economic evaluation, and consideration of social values – furnish the rationale for public drug funding recommendations by most HTA institutions. Most stakeholders found each of these components wanting in respect of child health and illness; many suggested creative ways to better calibrate them to assess drugs for paediatric indications.

Participants uniformly described intrinsic limitations to the production and interpretation of clinical evidence in children. Frequent comparisons to the generation of evidence for adult health technologies set these limitations in sharp relief. The poor fit to child health of accepted valuations of medical evidence surfaced repeatedly. Participants emphasized the difficulties of producing ‘high quality’ evidence in children in terms of current HTA standards – in one participant’s words, “the ability to generate evidence in a paediatric population that is ‘robust enough’ to answer questions” (HEA3). These difficulties were traced to both disease and population dynamics. The issues of trial availability and size predominated. Participants spoke about inherent challenges to recruiting trial subjects in the context of most childhood diseases, noting small numbers of affected children and historical reticence to involve children in clinical research. They related this reticence to an admixture of societal, professional, and industry concerns.

Safety was a major preoccupation in this regard. A lower societal tolerance for risk among children was felt to animate decisions – both at the level of design and enrolment – about children’s involvement in clinical trials:

*“I think from the societal level down to policy-makers, and even people on the front line in health care, there's a sense that in terms of the safety/efficacy balance we need to be more sure with respect to children than adults.”* (HEA1)

Diverse stakeholders identified the need for more and better paediatric drug safety data, in both pre- and post-market phases. A number identified the existence of provisions in other jurisdictions – notably the US and Europe – to both incentivize and compel industry to generate safety and efficacy data for paediatric indications of drugs new to market; they lamented the lack of comparable provisions in Canada. The perceived upshot of this relative lack of data on paediatric drug safety and efficacy is that “*you wind up adopting an adult intervention as the paediatric standard of care, without really the knowledge and the experience to do that safely and well*.” (HEA4).

In addition to upstream issues related to trial access and lack of data to support paediatric drug indications, participants identified downstream safety concerns attached to novel drug use in children. Most participants used the trope of ‘late effects’ to encapsulate this idea: the cumulative, often latent, and typically injurious side effects of a given treatment that accrue over the life-course. As participant responses evince, the potential effects of ‘late effects’ on drug access cut both ways. They can serve as putative barriers to the adoption of novel health technologies for children, inasmuch as lack of solid long-term safety data in children prompts deference to precautionary principles:

*“If you commit an 85-year-old to a drug that affects the bone marrow it has different implications to if you submit a 5-year-old to that, especially if it’s a condition for which they will need treatment for many years.”* (HEA6)

Conversely, factoring in a drug’s long-term effects can buttress arguments in favour of health system adoption, if the expected toxicity profile is promising relative to existing standards of care. However, it is the absence of confirmatory data on their real effect, coupled with the higher normative bar set for proof of safety in children, that participants viewed as a principal evidentiary barrier in current HTA approaches to child health technologies. The fact that many novel drugs in the era of precision medicine hold promise to reduce toxic side effects, both immediate and long-term, was seen by many as both a potential boon to children, and a factor inadequately recognized by current HTA paradigms.

Separately and together, these dynamics were identified as barriers to the generation of high quality evidence on the efficacy of drugs and other health technologies in children. Participants from clinical and HTA backgrounds acknowledged the integral role of randomized control trial (RCT) evidence of efficacy in current HTA paradigms, and emphasized the resultant challenges faced in assessing the worth of child health technologies. The risk of missed signals of clinical efficacy within traditional trial designs was of particular concern to those engaged in understanding the relevance of novel drugs or technologies in children:

"*When you have children who are being put into very specific baskets of their genomic and genetic markers and being treated for those…this problem of the uncertainty of the evidence is going to really hit an apex. It's going to mean looking at clinical trials and looking at evidence, and trying to figure out what is appropriate evidence, very differently. We're going to have to see a major, major shift*." (PAR4)

To many, the mismatch between epidemiological realities in children and the epistemic demands of HTA justifies a rethinking of our received appraisals of medical evidence. In the words of one participant, “*small numbers, small bodies, long outcomes: there has to be a different perspective*.” (HEA2) This recognition prompted many stakeholders to weigh and advance evolving notions of evidence in child health, and to catalogue novel approaches to generating evidence on child health technologies. Across the stakeholder groups interviewed, a nuanced sense of the legitimacy of diverse forms of evidence emerged: one that valued statistical confidence but placed greater emphasis on clinical and social context for its interpretation than is routinely exercised in received biomedical paradigms*.* This idealism was tempered by fidelity to rigour in evidence appraisal and the desire for reliable barometers by which to assess it. Related to this, participants acknowledged that the pace of institutional change would likely slow fundamental shifts in the norms attached to clinical evidence in existing HTA paradigms.

Nevertheless, most argued that innovative ways to produce, incorporate, and assess clinical evidence on paediatric drugs and other health technologies will prove essential to HTA institutions charged with their review. Many pointed to existing instances of this innovation, and opportunities to promote and formalize it. Recurrent themes included novel trial designs, such as genomic basket trials and n-of-1 trials; standardized guidelines for the use of historical comparators, where randomized exposures are infeasible or unethical; and evidence-building programs that adjudicate contingent funding approvals on the strength of accrued real-world evidence of effectiveness. Interestingly, many participants spoke to child health as an optimal testing ground for such innovation, because it is both crucial and tractable. As one parent put it, “*the problem is small enough, and the problem is big enough*.” (Parent) The need for new ways to measure and adjudicate the value of health technologies for children is critical, given the aforementioned limitations; at the same time, the population is both sufficiently bounded and prized to permit focused investment in this regard. A range of stakeholders characterized paediatric trials as pilot opportunities for approaches to evidence generation that will prove essential to adult drug development in an era of precision medicine. The necessity of such innovation in the paediatric space was seen as a key driver in this regard:

“*The numbers are smaller and so while that can be a disadvantage in terms of drug development, it can be an advantage in terms of being innovative in how you approach things. I think we’re starting to change the way we’re approaching the kids. How we change the way the system responds to them is going to be next*.” (PAR2)

In an emerging ‘golden age of new therapies’, a strong sense of the need for, and reality of, changing philosophies of treatment emerged. Participants viewed this reality as the foundation for new conceptions of the relative value of alternative forms of medical evidence, and their use in health technology assessment for children.

#### Economics

Often closely linked to their reflections on evidence, participants gave thoughtful and wide-ranging critiques of current modes of economic evaluation employed in dominant HTA paradigms. These insights spanned methodological and political challenges to the conduct and application of economic evaluation in HTA and drug policy for children. Uncertainty was a frequent trope invoked to relate evidentiary limitations in childhood illness and treatment to HTA constraints. Participants highlighted the implications for economic methods standardly employed in HTA, and the need to adapt them to deal with more and different kinds of uncertainty. The issue of altered time horizons, and the capacity to account for treatment-related health states along them, received particular focus:

*“[What] makes it very difficult is the time horizon, right? So, you know, often the economic models that we or others develop are explicitly for 5 years, or something like 5 years, and with kids, that may not be long enough to show the true effect of the intervention, and so you can of course produce models with a longer time horizon, but if you do that, then the uncertainty is always much higher.”* (PRO1)

Those interviewed enumerated challenges in accounting for both the positive and negative implications of therapeutic interventions across the life-course. The latent or chronic side effects of treatment, and their deleterious impact upon health-related quality of life at various life stages, were considered alongside potential gains in economic productivity from the combination of improved disease outcomes and less toxic therapy. Participants spoke to current limitations in measuring or modelling both, and the need to do better in the context of child health.

A range of stakeholders also identified limitations in the current tools used to gather and analyze data for the economic evaluation of health technologies for children. The relative merits and deficiencies of the quality-adjusted life year (QALY) as a core metric were front and centre. Participants gave special emphasis to inadequacies in current methods for eliciting preferences and assigning value to varied health states in children. One noted the temporal challenge of accounting for “*the dynamics of preferences in a developing organism*” (HEA1), wherein children at different development stages might assign widely differing value to similar health conditions. A number questioned the legitimacy of parental proxies in health state valuation for children, citing evidence for discrepancies in parent-child assignations of value to the same condition or intervention. Many spoke to the need to broaden the context for determining the value of child health states. Some cited emerging efforts to incorporate impacts on the utility of family members in a more holistic, family-centred measure of utility. Others noted an intrinsic prejudice against chronic morbidity in QALYs, one that could tend to devalue long-term health gains in children with chronic illnesses, and thereby privilege cure above disease maintenance, with insufficient consideration of other potentially relevant population characteristics, such as age, socio-demographics, or unmet need.

Conversely, a cross-section of participants referenced the manner in which QALYs favour children, in terms of accounting for potential life years gained by a given intervention. They framed investments in child health, and associated life years gained, as a return on investment, and articulated a narrative centred on the social and economic benefits reaped by such investments. A subset of participants intimated that the accounting of QALYs should incorporate sensitivity to differential benefit attached to health gains in critical developmental periods, in their capacity to condition the potential for gains in later years. Citing research on the economic returns from investments in early childhood development, these participants argued current approaches to both weighting and discounting QALY take insufficient account of the role of child health in social and economic potential across the arc of a life.

In light of the aforementioned challenges, a number of participants saw a crucial need for enhanced child health expertise in the economic evaluation of paediatric drugs and technologies. They related the capacity for nuanced and contextual understanding of the epidemiologic, developmental and sociological features of childhood as integral to legitimate exercises in health economic evaluation for child health interventions. Such perspectives linked participant views on economic evaluation back to their evidentiary concerns, and to their reflections on the political and sociocultural dimensions of policymaking on drugs and technologies for children, which we explore in further detail below.

#### Integration

The concept of *integration* encompasses participants’ reflections on the big-picture aspects of HTA production: firstly, its overarching framework and process; secondly, its interface with the surrounding health system. The interviews for this study occasioned frequent reflection on the optimal form and function of current HTA frameworks for the assessment of child health technologies. Some of these reflections offered subtle tweaks to existing HTA paradigms; others presented foundational critiques. Almost all participants spoke to the importance of a transparent and defensible framework as a core legitimator of a given HTA body and its recommendations. Most also referenced the key role of deliberative process in establishing optimal function, stressing the importance of procedural equity above allocative equality – ensuring a fair process first and foremost. Notably, the value of legitimate means was seen by some to outweigh any specific end:

“*There are some [decisions] that are black and white, and those ones should probably be reproducible. But there are many that are grey…if you had a different day, with a different composition of panel members, they might have voted the other way. The process should be transparent, it should be one where people articulate their reasons for the decision, but I don't know whether it always has to be the same*.” (POL7)

This concern for appropriate framework and process frequently dovetailed with perceptions of inadequacy in respect of children. The routine extrapolation of adult recommendations to the paediatric space was seen as a cardinal example of this. Some rendered this a specific instance of the fallacy of decision-making based on population averages. Others identified a distinct and more fundamental problem in the transposition from adult to child: the possibility that, due to differences in biology and social position, adult and child population distributions cannot be superimposed, let alone equated. In recognition of this, many defended the need for an HTA framework tailored to children. Their proposals in this regard incorporated sensitivity to aspects of child life and health such as developmental trajectories, future potential, and family context:

“*An unhealthy child is generally an unhealthy mother and, not uncommonly, an unhealthy father and siblings as well. So, the notion of unit of analysis, I think, is very germane to childhood*.” (HEA2)

The challenge of, and opportunity for, integrating alternative forms of evidence in any framework for child HTA was a paramount concern. Stakeholders related this both to the aforementioned constraints in generating traditional ‘high-quality’ evidence in paediatrics, and to the need to capture socially relevant dimensions of value not routinely represented in trial outcome measures. Some saw a role for enhanced patient and public engagement in this regard, including among children themselves. Related to this, a number of those interviewed spoke to the need for balance between structure and flexibility in child HTA guidelines, arguing that rigid form can stymie both the legitimate play of moral intuitions and the incorporation of scientific progress, including in HTA methods and processes:

"*You want guidelines that are going to be able to give people appropriate constructs to make decisions, but within those guidelines, you want to make sure they are not so hard and fast. Because the world – especially of paediatric cancer – is changing, and if you're creating guidelines which are too rigid that could be a real danger*." (PAR3)

A number of participants detected myopia in the frame typically applied to technology assessment, adult and child alike, arguing that *“[looking] at one drug at a time, in isolation from everything else”* (PRO1) gives a decontextualized – and therefore artificial – impression of the value of a given technology. The need to better incorporate system-situating factors like opportunity cost, unmet need, equity, and public priority was a recurrent motif. Many participants stressed the particular importance of broader context for the assessment of child health technologies, alluding to the unique play of evidence and social values in the paediatric space – for instance, the relevance of ideas like vulnerability and protection that often frame societal responsibilities to children as normative considerations to be weighed alongside clinical and cost effectiveness. Current approaches to incorporating social values in HTA for children were deemed especially weak.

From a process perspective, the critical need for paediatric expertise in the adjudication of child health technologies arose repeatedly. Participants identified this need at the level of both HTA institutions themselves and the drug funding bodies that receive and enact their recommendations. Interestingly, the push for paediatric representation in such fora was not made uncritically. A range of stakeholders recognized the potential for bias and agenda-pushing among child health experts tasked with assessing the value of a given drug or technology:

“*I think that group of pediatric experts needs to clearly carry the caveat you are not here to promote pediatric drugs. You are here to assess applicability, cost effectiveness, utility of drugs for pediatric purposes with the same rigor just with the perspective of what is possible on a pediatric evidentiary basis*.” (POL5)

However, this risk was often rendered as further proof of the need for a professionally and societally legitimate forum for child HTA: to limit opportunities for reactive or politically expedient decision-making on paediatric drugs and technologies, and to provide a structured forum to disentangle advocacy from dispassionate evaluation. While many participants were at some level invested in optimizing drug access for children, most also viewed objectivity as key to credibility in child-focused HTA. Rather than frame paediatric expertise as a barrier to objectivity, most saw it as a means of validating HTA for children.

Importantly, a few participants noted potential complexities that might arise from differentiated frameworks for adult and child HTA. They identified the transition from childhood to adulthood as particularly fraught, highlighting difficulties locating and justifying the switch from one state (and framework) to the next:

“*There is a transition from children to adults…for those conditions that are on the cusp, that affect both children and adults, and you have two separate processes, you can have different decisions, you know, someone turns eighteen, all of a sudden something's not available anymore*.” (POL1)

A few stakeholders also spoke to the points of overlap between HTA for children and rare diseases, noting that some of the provisions in rare disease frameworks are applicable to technologies for certain childhood diseases. However, most took care to point out that these commonalities did not erase distinct facets of child health that might warrant explicit incorporation in HTA frameworks. The challenge of when and to what degree special concerns related to child health – developmental vulnerability, future potential, family embedding, evidence constraints – apply within and beyond ‘child’ populations is not easily resolved. For most interviewed, these challenges did not diminish the relevance of such concerns to child HTA. They served instead as a cautionary note to unexamined paediatric exceptionalism, and as a means of sketching the defensible boundaries of a child-adapted HTA framework. Still, the potential for systematic bias in funding decisions arising from population-specific HTA frameworks – especially in view of the acknowledged challenge to incorporating opportunity costs into technology assessments – is a critical issue that any such distinct framework for child HTA will need to address.

The other major facet of technology ‘integration’ explored by study participants was the way in which HTA institutions interface with the health and social systems in which they reside. Many felt that current approaches to paediatric drug funding decisions in Canada are overly ad hoc, a compound result of: 1) inadequately sketched assessment parameters for children; 2) insufficient submission of paediatric technologies for formal review; and 3) politicized environments for the discharge of coverage recommendations. The role of emotion in stoking such politicization was front of mind for many:

“*All it takes is, you know, one article in the front page of a newspaper sometimes to make a minister change their mind*.” (PRO2)

Alongside this, however, a range of stakeholders suggested that a cultural and political shift is underway, toward the routine instantiation of HTA in policymaking on drugs and technologies at various health system levels in Canada. Many saw in this shift the potential to limit caprice in funding decisions. The opportunity for tightened and more effective interface between the various institutional players involved in drug access for children was a recurring theme. Innovative approaches to integrating clinical trial design and HTA processes were alluded to by a number of participants. For example, an adaptive pathways approach to drug development and access – which involves staged approval based on iterative, real-world evidence development and upfront involvement of patients and HTA bodies – was referenced as a means of creatively surmounting the data constraints attached to paediatric drug development*.* A few also pointed to the need for more and better-articulated HTA processes at the level of individual health care institutions. These stakeholders envisioned opportunities to make smarter and fairer choices among technologies through the application of HTA to resource allocation at the meso- and micro-levels, including global hospital and regional program budgets – particularly where such budgets are a priori allocated to child health.

By contrast, a number of stakeholders pointed to institutional reticence to conduct HTA in children – “*people often say, let’s not go there if we have to, like, let someone else work it out*” (POL1) – given both the methodological complexities and political sensitivities involved. This reticence was identified at various levels of the health system, from formal HTA bodies to hospitals to discrete disease-based programs. Of note, a range of participants laid at least part of the responsibility for the lack of volume and sophistication of paediatric HTA at the feet of the child health community itself. They identified a general lack of knowledge about HTA, a reticence to engage in political dimensions of health care, and an idealized view of children’s right to access health technologies as plausible culprits in the underdevelopment of HTA in the child health space. Even so, many perceived an opportunity to employ paediatrics as a rich testing ground for HTA development and reform, citing the strength of the child health community as a bounded and collaborative learning environment.

But the predominant theme on health system integration centred on its absence. Most participants felt that broader health system dynamics are only weakly considered in standard drug and technology reviews. They pointed in particular to the opportunity costs of investment in a given health technology or service, and lamented the absence of their routine incorporation in HTA recommendations and priority-setting endeavours. Many participants discerned the need for more holistic approaches to system-level planning in the face of resource scarcity, and the corollary need to reform HTA to enable such planning. Some felt this called for distinct funding pools for children, to compare like with like when gauging opportunity costs and to protect streams of funding for marginal populations. Others saw natural opportunities to enhance public funding for child health technologies, to which favourable budgetary and health impact profiles often attach. Most felt that, until HTA paradigms begin to take account of opportunity costs, their capacity to guide health systems toward choices that maximize societal value is limited. Once again, the need to better incorporate social values in HTA to guide such choices was voiced by the majority of respondents, and with it the obligation to attend thoughtfully to the unique considerations attached to children.

### Sociopolitical context

The social, political and economic determinants of drug access for children were front of mind for many participants. Their collective insights into the political and financial dimensions of drug development, marketing and regulation comprise a major category within the data. We generated three principal themes in this domain: *markets*, *governance*, and *politics*. Stakeholders ascribed foundational importance to these themes as the structural landscape within which paediatric drugs and health technologies are assessed for public coverage. Together, they were construed as the preconditions for HTA and the ultimate determinants of its impact.

#### Markets

Drug market dynamics were deemed by many participants as a principal determinant of HTA priorities and outcomes. A range of stakeholders made explicit links between drug development, assessment, and funding; the economic dimensions of drug production and sale were seen to motivate and shape these links. The role of industry figured prominently in this regard. Despite a clear need for novel paediatric drugs, weak market incentives for industry to engage in their research and development (R&D) were cited time and again by participants as an upstream determinant of drug availability for children. The narrow markets constituted by various paediatric disease cohorts were understood to limit the generation of evidence on drug safety and efficacy in children, with downstream impacts on the development of paediatric indications for novel and existing agents:

“*The market is extremely, extremely small … you're talking about very small, rare diseases, and/or short durations of therapy. So it creates difficulty in developing incentives on the regulatory side for pharma to develop products*.” (HEA4)

Participants construed the upshot of narrow markets as either industry disinterest in paediatric drug development or pricing tactics to coax profits from limited market space. Such tactics were often seen as illustrative of a fundamental misalignment of corporate and societal goals. Most participants related the disincentives for paediatric drug production to disincentives for HTA submission by industry, noting that “*the work involved in an HTA submission, which is quite a lot financially, sometimes doesn’t even pay off for them to bother*”. (PRO1).

Conversely, some participants perceived changing industry calculus on paediatric drug development, driven by the impact of new scientific knowledge on market incentives. The example of cancer is illustrative: as research uncovers the fundamental genomic and molecular drivers of various malignancies, and drug developers leverage these insights to create agents targeted at these drivers, the hard divisions between child and adult markets soften. In place of distinct paediatric and adult cancers emerge cancers defined by their molecular signatures, which may transit across traditional age boundaries. Markets adapt, and industry interest accordingly. Interestingly, the direction of these changes was understood differently by different stakeholders. Some saw children as a ‘spin-off market’ (HTA Professional) from adults; others saw children as the opportunity for first forays into wider adult markets:

“*We're finding more acceptance, or interest, from drug pharma, from big pharma that never had any interest in pediatric cancer, in these diseases, because it gives them a potential opportunity as a window to then branch out and use them in the adult population*.” (HEA6)

A few stakeholders also identified the lack of ‘levers’ available to HTA bodies to inform drug R&D dynamics, and underscored the impact of this impotence on drug portfolios where market incentives are lacking or where evidence generation is fraught. Related to the need for enhanced feedback capacity from HTA to industry to direct drug R&D priorities, a number of stakeholders spoke to the potential for governments to take more responsibility for funding novel drug trials in children. They highlighted access barriers related to the often-clumsy interface between industry-funded trials and government drug policy: these included jurisdictional hurdles to accessing trial drugs, resulting in geographic and disease-based disparities in access.

Alongside this, participants discerned a rapidly evolving research-clinical interface, in which the pressure to incorporate promising technologies into clinical practice is driving new approaches to evidence appraisal, and heightened expectations for real-world uptake:

“*You can go from a plan of research to using [a technology] in a clinical way within months… Now, you're using what was meant to be a research study for clinical action. Totally different paradigm than the classic clinical trial*.” (HEA7)

For many, the upshot of such observations was a perceived onus on governments to facilitate system uptake through nimble and creative policies that marry technology development, assessment and funding. These and other proposed corrections to market disincentives relate to the broader manner in which governments seek to mould paediatric drug market dynamics through legislative provisions and regulatory oversight. We explore drug policy and regulation in more detail in the section that follows.

#### Governance

The manner in which structures and mechanisms of governance – of the health system, of drug development and marketing, of public resource allocation – influenced drug access for children constituted a dominant theme amongst participants. The most consistent area of focus in this domain related to governmental policy and legislation on paediatric drug development, licensing, and sale. Participants described a variety of barriers to childhood drug access in the Canadian context, and ascribed a number of them to deficiencies in the drug regulatory environment. These deficiencies were juxtaposed with examples of regulatory reform and innovation in other jurisdictions, including the US and Europe, where specific provisions to buttress R&D for paediatric drugs have sought to correct for market constraints.

Limitations tied to the nature and scope of paediatric formularies were front and centre for participants, evidence of fundamental shortcomings with the R&D-to-market axis for most paediatric agents. Participants described problems related to the development, testing, production, licensing, and sale of drugs for children, and traced many of these to the poor fit between intrinsic dynamics of childhood disease and treatment and market-based systems for pharmaceutical development. The widespread lack of formal paediatric indications for existing and emerging agents was emphasized as a glaring symptom of this problem – both as a driver of off-label prescribing for children and as an indicator of evidence gaps in paediatric pharmacology:

“*There are a gazillion drugs that are completely standard of care for paediatrics that have no licensed indication for paediatrics… We're always piggybacking and trying to alter an adult solution to an adult problem to suit our needs*.” (HEA4)

The fact that industry will routinely forego paediatric testing of already-developed medications with known clinical utility in children was seen as illustrative of the thorough lack of market incentives for paediatric drug development, and deep industry disinterest in the same. Participants highlighted resultant problems at all points along the research-to-market pipeline, among which are: lack of knowledge on paediatric drug safety, efficacy and dosing; inappropriate product formulation for children’s varying size and developmental needs; lack of industry licensing submissions and market entry for paediatric indications; and, as a result of the foregoing, lack of HTA submission by industry for most drugs used in or relevant to children.

Description of these problems was routinely followed by mention of opportunities for their redress through regulation, real-world examples of the same, and identification of Canada as a laggard in this regard. Provisions by the US Food and Drug Administration (FDA) and European Medicines Agency (EMA) to both incentivize and compel industry to undertake paediatric testing of drugs were referenced by participants as indicative of the need for and role of Canadian regulation in this space. They also highlighted the downstream health system inefficiencies that result from desultory paediatric drug submissions and approvals at the national regulatory level. Among those mentioned were the human resource burden associated with Special Access Program (SAP) requests to Health Canada and the inconsistencies in responses thereto.

Some participants saw opportunities to take this regulatory oversight further: beyond shaping industry R&D activity and into health system priority-setting endeavours on childhood drugs and technologies. They envisioned a role for the use of high-level paediatric evidence – in the form of clinical guidelines – as a guide for national HTA priorities: both as a means of prioritizing paediatric drugs for licensing and coverage assessment, and articulating high-value areas for further R&D. Echoing calls among certain stakeholders to better integrate HTA and drug development paradigms, these participants envisioned a more proactive role for HTA institutions in drug policy and regulation, and described a role for the federal government to play in stewarding this reform.

A second sub-theme of governance centred on the need to enhance system stewardship and integration with respect to paediatric drugs. A range of stakeholders described the regulatory landscape mediating drug access for children less as a system than a loose interaction of institutional silos:

“*The payers know what's happening on their level, the pharma [companies] know what's happening on their level, and the patients know what's happening on their level – but no one actually really understands what's happening in each other's worlds*.” (PRO2)

Many identified access disparities issuing from weak governance and system fragmentation. The challenge of balancing access to medicines with sound and legitimate resource stewardship was felt to be particularly susceptible to the vagaries of a fractured political system. Participants identified these fractures at all levels of the health system, and across various sectors with bearing on drug policy and governance. The perceived result was disparity in drug access for children by geography, disease, and socioeconomic status, as well as relative disparity as compared to adults:

“*The other challenge in the paediatric context for when we get that drug in, expensive or not, that drug still has to be funded out of the institution's global budget. There's no unique funding envelope for these types of agents for children*.” (HEA3)

The corollary need for better harmonization of drug funding policy among system players was emphasized by a number of participants. In addition to cross-institutional policy coherence and broad stakeholder buy-in, one of the most salient benefits of such harmonization was felt to be integration of the component parts of drug policy – along the continuum from development and production to licensing and funding. Importantly, select stakeholders argued that the goal of rationalized system structure and governance need not preclude varied policy choices along provincial lines. Indeed, a few contended that differing provincial contexts necessitated jurisdictional autonomy on drug coverage decisions:

“*Why is everyone looking for even-Steven? Equity doesn’t mean equal, right?...If BC needs to make a decision that addresses the needs of BC's population, in the context of the budget that they have and the means and the resources – if that's our federated model – then BC should have the right do that*.” (POL5)

Provincial budgetary priorities notwithstanding, the vast majority of participants detected opportunities to improve the ways that drug policy for children is made and implemented in Canada. They collectively described a role for enhanced system stewardship at the federal level, to knit together the invested stakeholders and the component part of drug access over which they have influence.

#### Politics

Closely tied to insights about drug system structure and governance were participant acknowledgements of the highly political nature of drug policy decisions, and the broader national and provincial political currents that buffet such decisions. A number of participants framed their reflections on the political dimensions of drug policymaking with a presupposition of children’s marginality to political processes. The relative lack of agency ascribed to children was thought by many to abet a relatively low priority for child health on policy agendas:

“*Right now kids aren't figuring, aren't in the picture… because relative to adults there's so few of them getting sick. They don’t run lobby groups, they don't vote, they don't make contributions to political parties, so they really don't have a voice*.” (HEA1)

As a corrective to this natural state of political disempowerment, cause-specific advocacy was felt to play a critical role in marshalling attention to drug access barriers faced by children and pressing for political solutions. A range of stakeholders spoke to the increasingly vocal and impactful role played by patients and their families in advocating to government for enhanced access to child health technologies:

*“[Patients] are a much more powerful lobby than they were 10, 15, 20 years ago. And so they're driving the bus, a little bit. They're not sitting at the back of the bus any more, they're sitting right behind the driver. And I think that's going to change the way that policy is developed*.” (HEA2)

Participants pointed to the HTA-to-policy trajectory a key nidus for such advocacy. They identified a set of conditions susceptible to influence by advocacy groups, both for better and for worse, at distinct points along this trajectory. At the stage of HTA reviews, participants noted structured points for patient and family input into drug reviews, and associated opportunities to enrich the social values tranche of HTA. Importantly, a range of stakeholders was skeptical of the value of this input in its current form, noting that the perspectives given voice are typically narrow and biased:

*“[Patient advocacy groups] inevitably have almost irreparable conflicts of interest, because they're usually funded by the manufacturer of whatever thing we're reviewing. Their ability to elicit the values of their entire community is not good, it tends to be small numbers of particularly vocal patients*.” (PRO4)

At the stage of HTA uptake into policy, stakeholders pointed to a number of opportunities for influence by advocates. They recognized that governments lack meaningful knowledge about drug access issues for children, that stories related to child drug funding are often hot-button, and that opportunities exist to leverage the political optics of such stories in favour of children’s interests. Conversely, a few of those interviewed noted that the logic of re-election cycles often serves as a disincentive to investment in priorities with delayed or long-term returns. As the most compelling arguments to fund childhood health interventions, including drugs, often centre on their life-course impacts, a number of participants were less sanguine about political commitment to child drug funding in the face of high upfront costs:

“*It's hard to make the case to government to make decisions that will be financially fruitful many years down the road. We're saying invest in kids now because they're going to cost more if you don't later on. But they might not be in power later on*.” (HEA5)

In recognition of the parochial and highly politicized nature of many current advocacy endeavours, a few participants suggested creative means to improve advocacy to better align individual patient and societal goals. A focus on teaching and promoting ‘advocacy for the cause’ – as opposed to advocacy to advance access to discrete technologies – was a notable example:

“*When I say advocating for the cause it’s advocating because we need to change the regulations. We've done it drug by drug, but taking the generic case to the government really hasn't been done adequately…* ” (PAR4)

Stakeholders also emphasized the role of the media in influencing governmental decision-making on paediatric drug funding, including the manner in which HTA recommendations are handled. They identified media impact on public perceptions about drug access for children, and cited examples of ‘public pressure’ influence on political decisions about specific drug coverage. Many saw these dynamics as detrimental to both HTA institutions and collective societal interests, in their circumvention of transparent, dispassionate processes for technology evaluation and resource stewardship:

“*The things [for which] the government tends to override our decisions, I've found anecdotally, are generally things we recommend not to list that they end up listing because of public pressure*.” (PRO1)

Such reactive governance was juxtaposed with the careful, laborious, and resource-intensive process of HTA to demonstrate the bounded role for scientific evidence in the public domain, and to emphasize the importance of colloquial evidence and political calculation in ultimate coverage recommendations. In light of this, various stakeholders affirmed the need for an explicit and reliable process for adjudicating the value of child health technologies – one that not only leverages the transparent and deliberative approach of existing national HTA reviews, but incorporates a child-specific evaluation framework into its assessments.

## Discussion

Our study reveals unique health system and sociopolitical issues related to child health technology production, evaluation and use. These differences span the major phases of HTA, from assembly to assessment to integration, and the surrounding sociopolitical milieu, from markets to governance to politics. Careful analysis of these differences sets in relief a number of substantive and procedural challenges related to current HTA paradigms in respect of child health (Table 1). Taken together, these findings suggest a need to rethink how HTA is structured and operationalized for child health technologies: from the design of its component parts to the way they fit together. Focused attention to change along the continuum of HTA production, and to the regulatory systems that precede and receive that production, could yield substantial improvements in the quality and relevance of HTA for child health technologies.

**Table 1:** Problems and potential solutions in relation to drug policy for children in Canada

Domain	Sample Problem	Potential Solutions
**HTA Paradigms**		
Assembly	Priority-setting: • Manufacturer-driven process, minimal incentive for paediatric submissions	Transition from ‘push’ to ‘pull’ system for technology assembly: • National priority-setting framework • Resources for public sector-initiated HTA submissions • Patient/public engagement on social values to guide priority-setting
Assessment	Evidence appraisal: • Structural barriers to RCT-level evidence in paediatrics	Innovative trial design and evidence appraisal: • Basket trials, n-of-1 trials • Real-world evidence and performance-based funding mechanisms • Child health expertise in HTA bodies
	Economic evaluation: • Weak incorporation of child-specific considerations (developmental trajectory, preference elicitation) in pharmacoeconomic models	Advancement of science on child health economic evaluation: • Child-centred preference elicitation, family utility generation • Research on public preferences for resource allocation to children, including inquiry into life-course QALY weights
Integration	• Poor integration across phases and sectors involved in drug regulation and reimbursement	• Adaptive pathways approach to drug development, market access, and iterative evidence appraisal • Ring-fenced funding for paediatric drugs and health technologies • Engagement with child health experts throughout drug life-cycle
**Sociopolitical Context**		
Markets	• Weak industry incentives for paediatric drug development and licensing	• Federal regulatory mechanisms to incentivize/compel development and submission of paediatric clinical data • Public funding to subsidize novel drug trials in children
Governance	• Lack of formal paediatric indications, widespread off-label prescribing • Vertical and horizontal system fragmentation	• Dedicated paediatric expertise and resources within federal regulator and HTA bodies • Improved system integration along pharmaceutical value chain • National framework for drug regulation and funding for children to drive policy harmonization
Politics	• Weak political voice for children • Politically charged funding environment	• Enhanced receptors for child and family perspectives in drug regulation and funding decisions • Child-specific HTA framework to drive transparency and legitimacy in paediatric technology assessments, and mitigate potential for politically motivated drug funding decisions

### Implications for research and policy

Our findings suggest a number of opportunities for reform of existing HTA and drug policy environments to increase their relevance and responsivity to child health needs in Canada. The importance of legitimate process through the sequential phases of technology production – assembly, assessment, and integration – was a key theme in this regard. The focus on process legitimacy related largely to the inclusion of voice: at each phase, having the right range of perspectives incorporated into decision-making was a priority for participants. The precise make-up of that ‘right range’ sometimes differed among stakeholders, but commitment to the careful inclusion of varied perspectives was consistent.

In the **assembly** phase, many participants identified a relationship between power, voice, and the prioritization of technologies for review, one which has tended to privilege industry interests. Reliance on a predominantly manufacturer-driven submission process has allowed market logic to dictate the framing and selection of health technologies for HTA, including at the national level in Canada. Given commonly weak market incentives attached to the development and sale of paediatric drugs, this reality has limited the presence and prioritization of child health technologies in HTA pipelines. The formal inclusion of child health scientists, practitioners, patients and, notably, broader publics into institutional priority-setting endeavours was seen as a potential corrective. Academics and HTA institutions in other jurisdictions have issued comparable calls for enhanced voice in HTA priority setting for both general publics and discrete constituencies within them [18–20]. The findings from this study affirm and amplify such calls. Importantly, they also direct attention to the impact of distinct voices on the prioritization of technologies, and highlight the need to attend to the natural disadvantage of child health technologies in standard HTA priority setting processes.

In the **assessment** phase of HTA, the improved elicitation of social values emerged as a crucial opportunity for procedural reform. Questions of whom to involve in defining and adjudicating value amongst competing child health technologies, and how to involve them, recurred across interviews. Cataloguing the range of problems with paediatric evidence generation and interpretation, and with methods for the economic evaluation of child health technologies, participants argued forcefully – and uniformly – that child health expertise is essential to meaningful technology assessment in this population. They saw viable nodes to incorporate such expertise in the appraisal of clinical evidence, and in the design and interpretation of pharmacoeconomic models for technology evaluation. At the micro-level, a number identified opportunities to strengthen the foundations of child health economic evaluation through methodological reform of preference elicitation for child health states, including the incorporation of familial impacts of child illness and treatment. At the macro-level, many participants endorsed the development of a coherent framework for child HTA that explicitly incorporates valuations of life-course potential alongside more generic considerations like unmet need. Lastly, participants stressed the importance of eliciting social values through deliberation with patients and publics. Future efforts to elicit societal priorities related to funding decisions for child health technologies could seek to incorporate children’s voices in deliberative processes and strengthen fundamental knowledge on societal preferences vis-à-vis resource allocation in children.

In the **integration** phase, process considerations were deemed central to technology uptake into the surrounding health system. The transition from HTA recommendation to policy was identified as a particularly weak joint in the evidence-to-policy continuum. Participants cited opportunities to mitigate the political vagaries impacting funding decisions for child drugs and technologies through investment in innovative trial designs, such as adaptive pathways, that incorporate staged drug approval, evidence of real-world safety and effectiveness, and input from HTA institutions, patients and publics across a drug’s life span [21]. Another critical issue is the need to consider opportunity costs in drug coverage decisions by comparing like with like. Some participants felt that a first, if imperfect, step toward this goal is distinct funding pools for paediatric drugs. At minimum, points of routine, structured engagement with child health communities, experts and patients alike, would help funders place HTA recommendations for specific paediatric technologies in broader system context.

Achieving policy coherence from this range of potential reforms implies the need for a national framework to guide drug policy for children, one that takes account of the distinguishing features of child health, illness, and treatment from drug development to coverage. Any such framework would need to focus on two principal aims: reform of the drug regulatory system to better incentivize paediatric drug research, development and product licensing; and development of nationally adopted drug funding guidelines for children. Attending to the unique sociocultural, scientific, and political dynamics that condition access to medicines for children – both within HTA institutions and beyond – would strengthen the technical and moral bases of coverage decisions, providing for sounder and more equitable access to new and existing paediatric drugs.

### Study strengths and limitations

To our knowledge, this study provides the first empirical evidence about the unique health system and sociopolitical dimensions of HTA for children. It integrates rich qualitative data from a diverse range of stakeholders to generate insights about how to understand and improve drug assessment and policymaking for children in public health systems.

Our findings are bounded by the scope of our sample, which examined the Canadian HTA and health system context, with specific focus on Ontario drug policy dynamics. Our capacity to account for Canadian cross-provincial differences in drug policymaking environments or uptake of national HTA recommendations is therefore limited. This limitation is mitigated by the increasingly national scope of drug assessments, funding recommendations, and policy uptake in Canada. The relevance of our findings to other health system contexts internationally is likewise open to question. Remarkably, almost no evidence exists on the principles and processes of drug funding decisions for children in any health system context. This study serves as a first in-depth foray into the unique considerations attached to HTA for children, with sufficient context-specificity to yield both foundational and particular knowledge for policy. Future work could focus on extending such system and policy analyses to cross-country comparisons of HTA for children.

Lastly, our study sample focused on a range of stakeholders involved in or impacted by drug coverage decisions for children in Canada, including HTA professionals, provincial policymakers, health professionals, and patient family members. We did not formally sample members of the general public, nor undertake purposive sampling of other segments of the population with unique drug access experiences, such as those with rare diseases. Lastly, we did not interview children themselves. Involving these voices in future analyses of child HTA may yield novel insights.

## Conclusion

Current approaches to health technology assessment are not well calibrated to the realities of child health and illness. Our study furnishes unique empirical data on the political and health system dynamics of drug funding decisions for children in Canada, relevant to both HTA institutions and government payers. It interrogates the fit of each phase of the HTA process – assembly, assessment and integration – to child health realities, and emphasizes opportunities to improve current approaches to the assessment of paediatric drugs and technologies. This produces a nuanced and contextually grounded analysis of concepts instrumental to drug funding decisions for children. The insights generated are directly applicable to the Canadian and Ontario contexts, but also yield fundamental knowledge about the normative dimensions of HTA for children that are germane to drug policy in other health systems.

## Supplementary information

**Additional file 1.**

## Data Availability

The datasets generated and analysed during the current study are not publicly available due the potential for participant identification, but are available in redacted form from the corresponding author on reasonable request.

## Abbreviations

CADTH: Canadian Agency for Drugs and Technologies in Health
EMA: European Medicines Agency
EU: European Union
FDA: Food and Drug Administration
HEA: health professional
HTA: Health technology assessment
PAR: Parent
PCPA: Pan-Canadian Pharmaceutical Alliance
POL: Provincial policymaker
PRO: HTA professional
QALY: Quality-adjusted life-year
R&D: Research and development
SAP: Special Access Program
US: United States
