# Supplementary material for: ‘The problem is small enough, the problem is big enough’: a qualitative study of health technology assessment and public policy on drug funding decisions for children
Source: Int J Equity Health. 2020 Mar 30;19:45. doi: 10.1186/s12939-020-01164-w (PMC7106721; doi:10.1186/s12939-020-01164-w)
Supplement: Supplementary file 1 — Additional file 1. [file 12939_2020_1164_MOESM1_ESM.docx]

# Semi-structured interviews: HTA professionals/clinicians/policymakers

**Discussion topics and interview guide**

INTERVIEW DATE/TIME: _____________________

**Pre-interview**

- Review purpose of study and why participant selected
- Review and collect informed consent
  - Review anonymity conditions
  - Review withdrawal rights (any point during/after conduct of interview)
- Describe nature/structure of interview

*[Begin recording]*

**Introduction**

- **State date**
- **State professional role:**
  - Politician/bureaucrat/policymaker
  - HTA professional
  - Clinician (physician, pharmacist)

***Preamble:***

- This study is one part of a larger project examining health technology assessment and drug policy for children in Canada. The focus of the project is trying to understand if our existing HTA processes and outputs meet the needs of children – that is, whether they are relevant to the realities of child health and health care in our country. In particular, the project seeks to understand whether the social values that motivate and structure HTA are, or should be, different with respect to the assessment of child health technologies.
- This particular study seeks insights from those involved in, or impacted by, HTA for child health technologies – clinicians, HTA professionals, and patient families – about the context for paediatric drug funding and access in Ontario, the values that predominate in that context, and the strengths and challenges that result.

***Research questions:***

- What social values are relevant to child HTA? What unique considerations should inform the appraisal and selection of child health technologies for public coverage?

**NOTESInterview Questions**

**Topic: Child health and novel therapeutics**

***Preamble:***

- One of the motivating factors for this study is the perceived increasing relevance of novel drugs and diagnostics in paediatric medicine, and the rising costs of the same, coupled with an acknowledgement that the wider context for drug research, development and regulatory environments often creates challenges in access to medicines for children.

***Questions:***

- What is your sense of the relevance of novel drug therapies in paediatrics? How do you think this compares to adult medicine?
- What are your perceptions about the current state of the drug development and regulatory pipeline for paediatric medicines in Canada?
- What challenges, if any, do you perceive in access to novel drug therapies for children in Canada?

***Alternatives for clinicians***:

- Have you experienced successes and/or challenges with obtaining access to existing or novel drugs that you thought would benefit your patients? Please describe.
- How, in your view, do the dynamics of access for children relate to drug licensing and/or funding in Canada?

**NOTES**

**Topic: HTA and access to paediatric drugs**

***Preamble:***

- The relationship between HTA and drug policy decisions is not 1:1. Sometimes HTA recommendations are not followed by policymakers; sometimes there are no HTA recommendations to follow, either because they are not initiated or are not feasible (e.g. due to lack of evidence or time).

***Questions:***

- What is your sense of the current role of HTA in drug access dynamics for children in Canada? What strengths and challenges do you perceive with current models of HTA for paediatric drugs and diagnostics in Canada?
  - Perceived relevance of existing HTA processes and methods to child health conditions and system realities in Canada
  - Perceived deficiencies and/or inefficiencies of such methods vis-à-vis novel drug assessment and funding for children in Canadian and Ontario context(s)

***Alternatives for clinicians***:

- Are you aware of HTA processes/institutions in Canada/Ontario? Do you think these impact your clinical milieu or care of patients? If so, how?
- In what ways, if at all, have provincial drug funding decisions impacted your care of patients?
- What is your sense of the relationship between HTA processes and provincial drug coverage decisions for children in Ontario?

**NOTES**

**Topic: Social values, health policymaking and child HTA**

***Preamble:***

- Social values may be said to be the shared moral sensibilities or intuitions that motivate or justify collective programs of action, be they social policies or programs, political endeavours, or that mediate the nature of relationships between individuals in a given society.
- In the context of HTA, certain ‘values’ have tended to predominate:
  - **certainty** or truth, founded on particular types of knowledge or evidence;
  - **value** for money, based on notions of efficiency; and
  - **feasibility**, gauged with reference to system and societal impact.

***Questions:***

- What values do you think are most important to HTA processes or outcomes? Are there important social values that you feel aren’t well represented in our current HTA paradigms?
- Are there any social values that you think should attach specially or more centrally to HTA for children?
- Do you think that the principle and/or processes for HTA for child health technologies – such as drugs, diagnostics and services – should differ from those for adult technologies? If so, why? How?
  - Are children owed different opportunities or protections when health systems adopt new technologies? Why or why not?
- Which stakeholders do you think are important to engage in drug funding decision-making in the province? Please describe.
  - (Perceptions of the respective roles of health care practitioners, HTA professionals, patients and the public in drug funding decision-making)
- What types of evidence/knowledge do you think are important to consider in the context of provincial public drug funding decisions? How, if at all, do you think the relevance of these sources of knowledge differ between HTA for adult and child technologies?
- What are you thoughts on the relative importance of clinical evidence, health system economics, and patient or public values in drug funding decisions and policy?
  - Potential differentiating features/dynamics in paediatric vs. adult populations
  - Potential differentiating features of child HTA and drug funding from rare disease/orphan drug dynamics and policies

**NOTES**

**Topic: Future directions**

- Given the discussion above, do you think we need to develop/incorporate different policies, programs or processes to guide child HTA or drug funding decisions, either provincially or nationally?

**NOTES**

**Post-interview**

- Thank participant
  - Parking costs
  - (+gift card for parents)
- Reiterate anonymity provisions
- Request consent to follow up with participant at later date for member checking, as needed

**NOTES**

**Appendix**

**Lay Summary of Program**

**Project Title:** The politics of child health technologies: Social values and public policy on drug funding decisions for children in Canada

**Project Summary:** Drug research, development and policy have historically neglected children. One area of persistent neglect is public policy on funding for paediatric medicines. In most publicly funded health systems, including Canada, decisions about which drugs to cover are made through a formal process called health technology assessment (HTA). This project examines the role and challenges of HTA as applied to children, with a focus on the role and substance of social values therein.

**Previous Research:** The vast majority of HTA research has focused on adult health problems and technologies. Child health has received little attention. There is growing awareness that HTA as currently conducted presents a variety of problems in the context of child health.

**Project Description:** Are children owed different opportunities and protections when health systems adopt new technologies? This project will explore how we assess the worth of child health technologies for public funding. We will conduct a mixed methods study of social values in child HTA, through case studies of recent paediatric drug funding decisions in Canada. Interviews with stakeholders involved in or impacted by HTA for child cancer drugs in Canada will inform design and conduct of a survey on social values for child HTA.

**Impact and Relevance:** Children are a distinctive test of the social values that influence our choice of health technologies for public coverage. This research will deepen our understanding of the assessment of child health technologies, and generate evidence on the social values that influence this process. Ultimately, such knowledge will help guide policy decisions on which drugs to cover for children and why.
